# Evidence of an anti-apoptotic effect of qinghuobaiduyin on intestinal mucosa following burn injury

**DOI:** 10.3892/etm.2013.1314

**Published:** 2013-09-26

**Authors:** JIE ZHU, PING WANG, QUANYONG HE, JIANDA ZHOU, CHENGQUN LUO

**Affiliations:** 1 Department of Burn and Plastics Surgery, Third Xiangya Hospital, Central South University, Changsha, Hunan 410013, P.R. China; 2 PET/CT Center, Hunan Provincial Tumor Hospital, Changsha, Hunan 410013, P.R. China

**Keywords:** qinghuobaiduyin, burn injury, apoptosis, intestinal mucosa

## Abstract

Burn injuries are common in wartime and in times of peace. The prevention and therapy of ischemia-reperfusion injury to the organs, in particular the intestine, during the burn shock and recovery process has become a popular yet challenging area of research. Studies concerning the apoptosis of the cells of the burned intestinal mucosa have gained considerable attention. Qinghuobaiduyin (QHBDY) is a traditional Chinese medicine that has been used as a clinical prescription since 1995 to treat burn patients due to its opsonization function in the immune system and favorable clinical therapeutic effect. The aim of this study was to investigate the effect of QHBDY on the apoptosis of intestinal mucosa following burn injury. An animal model was constructed comprising severely burned rats that were treated with various dosages of QHBDY. Tissues from the small intestine were collected to investigate the apoptosis rate by TUNEL assay and the protein expression levels of heat shock protein 70 (Hsp70) and caspase-3 by immunohistochemistry. In addition, IEC-18 cells treated with QHBDY and burn serum were investigated. The cell apoptosis rate was analyzed by flow cytometry (FCM), the protein expression levels of Hsp70 were measured by western blot analysis and caspase-3 activity was analyzed by a colorimetric assay. The results showed that in animal experiments, compared with the burned group, the apoptosis rates in the treatment group was decreased, the protein expression level of Hsp70 was increased while Caspase-3 was decreased. In cell experiments, after treatment with QHBDY, the cell apoptosis rate was lower than that of the burn serum group. In addition, Hsp70 protein expression was upregulated and caspase-3 activity was decreased. QHBDY may play an important role in the prevention of apoptosis at the whole animal and cellular levels.

## Introduction

Burn injuries are frequent in wartime and also in times of peace. The prevention and therapy of ischemia-reperfusion injury to the organs, particularly in the intestines, during the burn shock and recovery process has become a challenging focus of research. The mucosal cells are an important component of the intestinal mucosal barrier structure. A previous study has shown that the apoptosis of mucosal cells is the main form of cell death occurring with intestinal ischemia-reperfusion, with apoptotic cells accounting for 80% of the total number of dead cells (1). Therefore, studies concerning the apoptosis of cells in the burned intestinal mucosa have gained an increasing amount of attention.

The heat shock protein 70 (Hsp70) family is the most conservative and important category of HSPs. Hsp70 is abundant in the majority of organisms, and is generated most significantly during cellular stress; therefore, Hsp70 has been studied the most extensively and in-depth (2). Hsp70 plays an important role in the protection of the gastrointestinal mucosa and previous studies have shown that Hsp70 has a variety of important physiological functions (3–7), including acting as a chaperone, cytoprotection, anti-apoptotic and anti-oxidation functions and participating in the immune response.

Caspase-3 is a key enzyme in the caspase family that is activated by various apoptosis-stimulating factors. Activated caspase-3 could induce apoptosis via acting on other members in caspase family (8). Caspase-3 may directly cleave Bcl-2 into fragments, so that the function of Bcl-2 is changed from the inhibition of apoptosis to the triggering of apoptosis (9,10). The role of caspase-3 in ischemia-reperfusion-induced apoptosis is a topic that is gathering increasing interest.

Burn scholars have proposed and developed a variety of treatment options that have achieved certain therapeutic effects. However, for a variety of reasons, including lack of functional diversity, unstable efficacy, toxicity and high cost, these treatments have been challenging to apply in the clinic.

Qinghuobaiduyin (QHBDY) is a traditional Chinese medicine that contains *Astragalus membranaceus*, *Lonicera japonica*, *Scutellaria baicalenis* Georgi, *Ophiopogon japonicus*, *Rheum rhabarbarum*. QHBDY has been used as a clinical prescription since 1995 to treat burns due to its opsonization effect on the immune system and favorable clinical therapeutic effects (11,12).

Several studies have shown that certain components of QHBDY have anti-apoptotic effects in certain tissues and organs. The aim of the present study was to investigate the protective effect of QHBDY against apoptosis of the intestinal mucosa.

## Materials and methods

### Materials

The QHBDY was prepared by the Chinese medicine laboratory of The Third Xiangya Hospital of Central South University (Changsha, China). The caspase-3 colorimetric assay kit and Annexin V-FITC and propidium iodide (PI) apoptosis assay kit were purchased from Kaiji Biotech (Nanjing, China). The antibody against caspase-3 was obtained from Cell Signaling Technology, Inc. (Beverly, MA, USA). The anti-Hsp70 antibody was purchased from Seajet Scientific Inc. (Beijing, China). The anti-GAPDH antibody was from Santa Cruz Biotechnology, Inc. (Santa Cruz, CA, USA). The cell culture media and reagents were obtained from Invitrogen (Carlsbad, CA, USA). The IEC-18 cells were from American Type Culture Collection (ATCC; Baltimore, MD, USA). The Sprague Dawley (SD) rats were provided by the Experimental Animal Center of The Third Xiangya Hospital of Central South University. Both normal and burn serum were collected from the rat heart.

### Model construction of severely burned rats

The animal experiments were approved by the Institutional Animal Care and Use Committee of Central South University. The methods for the model construction of severely burned rats were previously described by Walker and Mason (13). Briefly, 136 healthy SD rats weighing 180–220 g were divided into three groups randomly: normal group (n=8), burned group (n=32) and treatment group (n=96). According to the treatment dosage of QHBDY, the treatment group was subdivided into groups of 0.5 ml/100 g, 1 ml/100 g or 1.5 ml/100 g (n=32 for each group). Under anesthesia, the nude skin was scalded with a 97°C water bath for 18 seconds to cause a 3rd degree burn encompassing 30% total body surface area (TBSA). The rats were resuscitated with an intraperitoneal injection of Ringer’s lactate in the volume of 4 ml/kg/1% TBSA, placed in individual cages and allowed free access to food and water. The normal group had the same procedure with the exception that the water temperature was 37°C. The rats in treatment group were given 1.0 g/l QHBDY by oral gavage twice within 24 h before burning. After burning, 1.0 g/l QHBDY was administered again within 5 minutes. The burn area calculation and control was performed according to the rat’s total body surface area with the formula: Area (cm^2^) = K × W^2/3^, W is the weight (g), K=10. The desired burn area was 30% of the TBSA.

### Cell culture

IEC-18 cells were maintained in Dulbecco’s modified Eagle’s medium (DMEM) supplemented with 10% fetal bovine serum at 37°C in a humidified atmosphere of 5% CO_2_. Serum and QHBDY were diluted with 1% saline solution (vol/vol) into appropriate concentrations, which were then added to treat cells for 24 h

### Western blot analysis

Monolayers of IEC-18 cells were washed twice with ice-cold phosphate-buffered saline (PBS) and lysed by ice-cold lysis buffer [150 mM Tris-HCl (pH 7.4), 150 mM NaCl, 1% Nonidet P-40, 2 mM EDTA, 50 mM sodium fluoride, 0.2% SDS, 100 mM sodium vanadate and 1 mM phenylmethylsulfonyl fluoride]. After 30–60 min on ice, lysates were cleared of cellular debris by centrifugation (13,000 × g) for 1 min at 4°C. Protein (50 μg; determined by the Bradford protein assay) was diluted in 5X SDS-PAGE sample buffer [150 mM Tris base (pH 6.8), 30% glycerol, 4% SDS, 7.5 mM dithiothreitol (DTT) and 0.01% bromophenol blue] and separated on 10% SDS polyacrylamide gel. Following electrophoresis, the separated proteins were transferred to nitrocellulose (NC) membranes. The membranes were incubated overnight in 5% nonfat milk in Tris-buffered saline containing 0.1% Tween-20 to saturate the nonspecific binding sites. The membranes were then incubated with anti-Hsp70 primary antibody (dilution, 1:400) for 1 h at 37°C. The protein bands were visualized using horseradish peroxidase-conjugated secondary antibody (Goat Anti-Rabbit IgG, Santa Cruz) with a chemiluminescence-based detection system (ECL western blotting kit; Pierce Biotechnology, Inc. Rockford, IL, USA). For molecular weight determinations, multicolored protein markers were used. To verify equal protein loading, membranes were stripped and reprobed with anti-GAPDH monoclonal antibody (dilution, 1:1,000). The intensity of protein bands was quantified using a ScanJet 4C Flatbed Scanner (Hewlett-Packard, Palo Alto, CA, USA) with NIH Image v1.52 software (http://rsb.info.nih.gov/nih-image/). Lightly exposed films were used for quantification.

### Immunohistochemistry

The tissues from the small intestine were fixed in 10% paraformaldehyde solution, then embedded in paraffin or frozen. The sections (4-mm thick) were cut from tissue blocks and mounted on slides, then sections were baked at 60–65°C for 4 h. The slides were incubated in xylene and graded ethanol to remove the paraffin. Antigen retrieval was performed with a high temperature and high pressure citrate buffer. Goat serum was used to block nonspecific staining and H_2_O_2_ to quench endogenous peroxidase activity. The slides were then incubated with primary antibody (caspase-3, 1:500; Hsp70, 1:500) for 1 h at 37°C. After washing with PBS, the secondary antibody (Goat Anti Rabbit IgG-HRP, Maixin, Inc., Fuzhou, China) was added and the slides were incubated at 37°C for 1 h. The staining was visualized using a DAB staining kit (Maixin, Inc.) according to the manufacturer’s instructions and samples were counterstained with hematoxylin and eosin (H&E) before viewing under a microscope (Nikon, Tokyo, Japan). Under high-magnification microscopy, five visual fields were randomly selected to assess the optical density of positively immunostained cells. The average gray value was then calculated for quantitative analysis.

### TUNEL assay

The tissue samples were fixed in 10% paraformaldehyde solution for 24 h, then embedded in paraffin. The paraffin-embedded tissues were cut into 4-μm sections. TUNEL assays were performed using the In Situ Cell Death Detection kit (Kaiji Biotech) according to the manufacturer’s instructions. The number of apoptotic cells was counted under an optical microscope.

### Caspase-3 activity assay

The caspase-3 activity assay was performed using the caspase-3 activity assay kit according to the manufacturer’s instructions.

### Flow cytometry (FCM)

For measuring the apoptosis rate, cells were dual-stained with PI and Alexa Fluor 488-Annexin V using an Annexin V-FITC and propidium iodide (PI) apoptosis assay kit (Kaiji Biological Inc.) according to the manufacturer’s instructions. The stained cells were analyzed by FCM (FC 500 MPL system; Beckman Coulter, Inc., Miami, FL, USA).

### Statistical analysis

The data were analyzed for statistical significance using a Student’s t-test. P<0.05 was considered to indicate a statistically significant result.

## Results

### Effect of QHBDY on the mucosal cell apoptosis rate in the small intestine in burned SD rats

**Figure 1:**
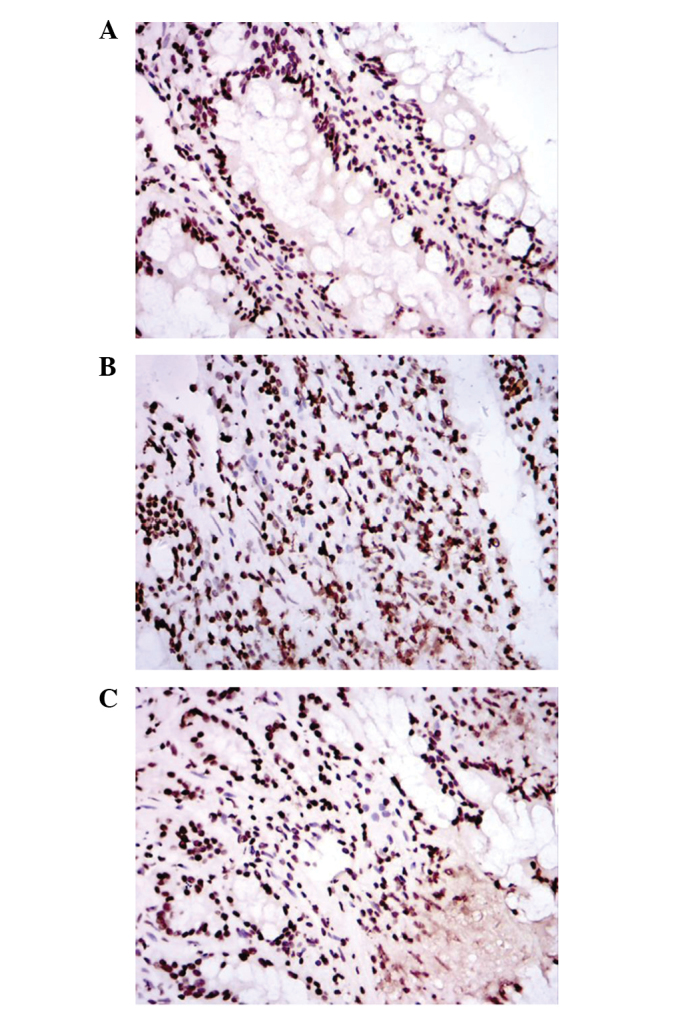
Apoptotic cells in small intestinal mucosa revealed by TUNEL assay. The nuclei of apoptotic cells were brown and the apoptotic cell number in treatment group was decreased as compared to normal group. (A) Burn group, (B) 1 ml/100 g QHBDY group and (C) 1.5 ml/100 g QHBDY group. QHBDY, qinghuobaiduyin. Magnififcation, ×400.

**Figure 2:**
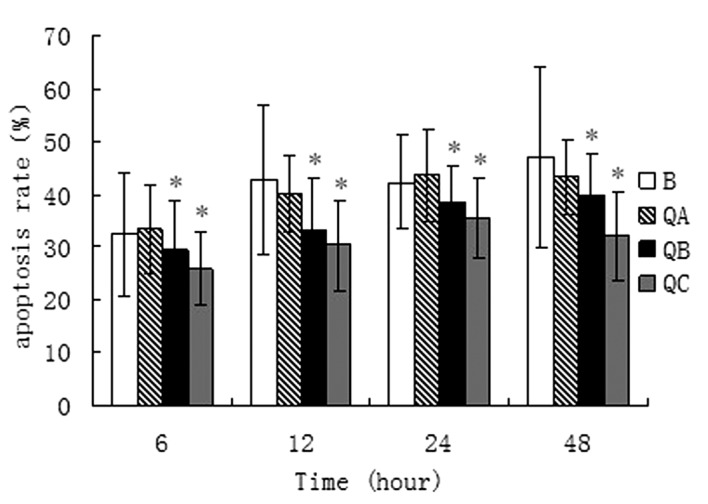
Effect of qinghuobaiduyin (QHBDY) on the apoptosis rate of the small intestinal mucosa. Data are expressed as mean ± SD. ^*^P<0.05 compared with group B (burned group). QA group, 0.5 ml/100 g QHBDY; QB group, 1 ml/100 g QHBDY; QC group, 1.5 ml/100 g QHBDY.

The burned SD rats in the three treatment groups were treated with QHBDY at dosages of 0.5 ml/100 g (QA group), 1 ml/100 g (QB group) and 1.5 ml/100 g (QC group), and the apoptosis rate of the mucosa of the small intestine was observed at time points of 6, 12, 24 and 48 h (Fig. 1). As shown in Fig. 2, the apoptosis rates in the treatment groups were lower than in the burned group (B group), and the apoptosis rates in the 1 ml/100 g and 1.5 ml/100 g groups were statistically different from that of the burned group (P<0.05).

### Effect of QHBDY on Hsp70 expression at the protein level in the small intestines of burned SD rats

**Figure 3:**
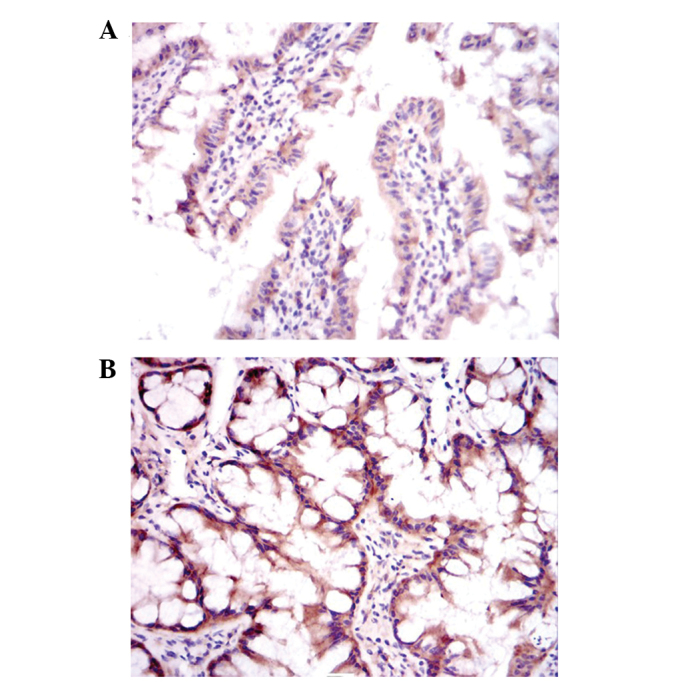
Immunohistochemical staining of Hsp70 in small intestinal mucosa. Cells with Hsp70 positive staining had brown staining in cytoplasm and nucleus, the staining in the 1.5 ml/100 g group was stronger than the burn group. (A) Burn group and (B) 1.5 ml/100 g group. Hsp70, heat shock protein 70. Magnififcation, ×400.

**Figure 4:**
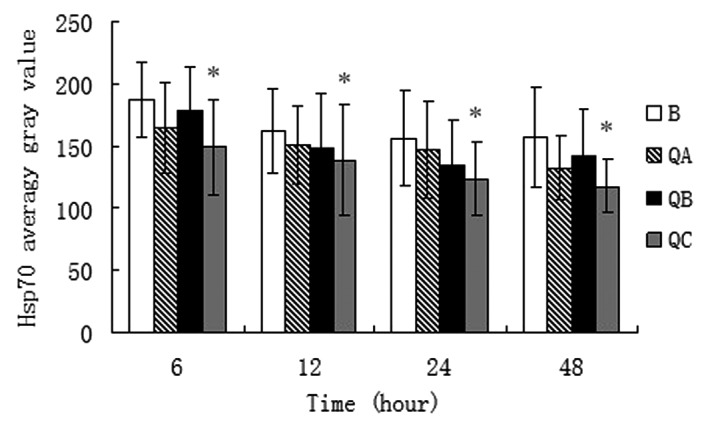
Effect of qinghuobaiduyin (QHBDY) on Hsp70 protein expression in small intestinal tissue. Data are expressed as mean ± SD. ^*^P<0.05 compared with group B (burned group). QA group, 0.5 ml/100 g QHBDY; QB group, 1 ml/100 g QHBDY; QC group, 1.5 ml/100 g QHBDY. Hsp70, heat shock protein 70.

The burned SD rats in three treatment groups were treated with QHBDY at dosages of 0.5, 1 and 1.5 ml/100 g. Tissues were collected from the small intestine following treatment times of 6, 12, 24 and 48 h for immunohistochemical staining (Fig. 3). The expression level of Hsp70 protein was measured using the analysis software of the microscope. As shown in Fig. 4, there was a statistical difference in the level of Hsp70 protein expression between the 1.5 ml/100 g treatment group and the burned group (P<0.05) at 6, 12, 24 and 48 h. However, no differences were identified between the other two treatment groups and the burn group.

### Effect of QHBDY on caspase-3 expression at the protein level in the small intestines of burned SD rats

**Figure 5:**
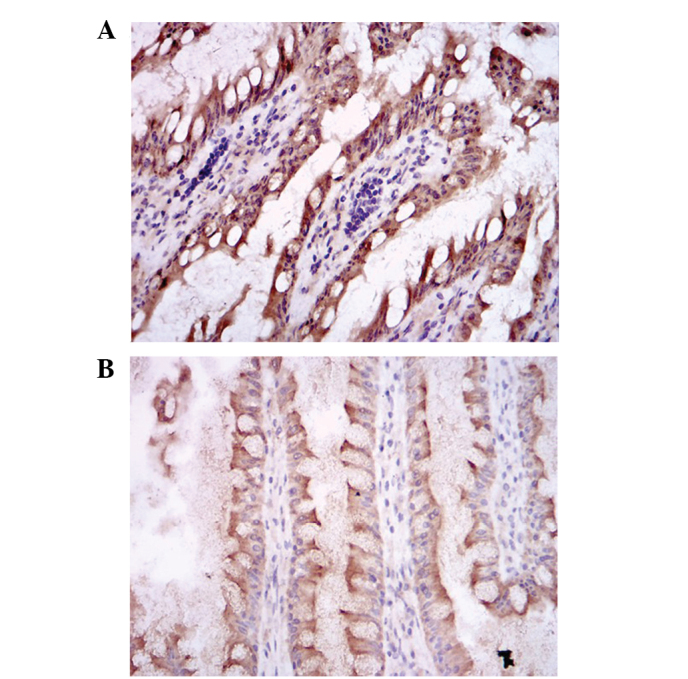
Immunohistochemical staining of caspase-3 in the mucosa of the small intestine. Cells with caspase-3 positive staining had brown staining in the cytoplasm, the staining in the 1.5 ml/100 g group was weaker than the burn group. (A) Burn group and (B) 1.5 ml/100 g QHBDY group. QHBDY, qinghuobaiduyin. Magnififcation, ×400.

**Figure 6:**
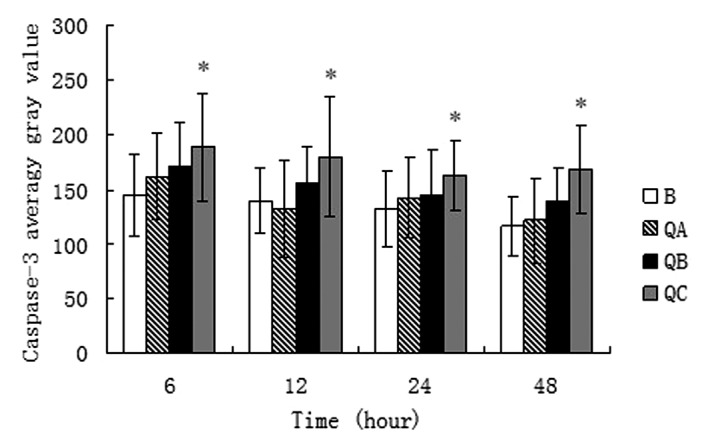
Effect of qinghuobaiduyin (QHBDY) on caspase-3 protein expression in tissue from the small intestine. Data are expressed as mean ± SD. ^*^P<0.05 compared with group B (burned group). QA group, 0.5 ml/100 g QHBDY; QB group, 1 ml/100 g QHBDY; QC group, 1.5 ml/100 g QHBDY.

At 6, 12, 24 and 48 h after the treatment of the burned SD rats with QHBDY at three different dosages, the tissues were collected from the small intestine to investigate the effect of QHBDY on caspase-3 expression (Fig. 5). The results show that QHBDY at the dosage of 1.5 ml/100 g was able to decrease the expression of caspase-3 following treatment for 6, 12, 24 and 48 h compared with that in the burned group (Fig. 6).

### Effect of burn serum on caspase-3 activity and IEC-18 cell apoptosis rate

**Figure 7:**
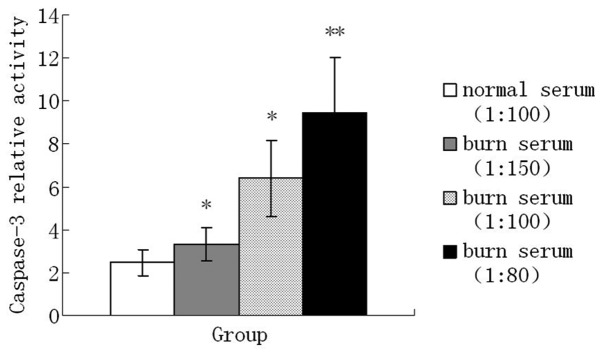
Effect of burn serum on caspase-3 activity in IEC-18 cells. Data are expressed as mean ± SD. ^*^P<0.05 and ^**^P<0.01 compared with the normal serum group.

In order to study the effect of burn serum on caspase-3 protein activity, burn serum was added to IEC-18 cells for 24 h at three different concentrations (1:150, 1:100 and 1:80), or normal serum (1:100) was added to IEC-18 cells as a control. As shown in Fig. 7, following treatment with burn serum, the relative activity of caspase-3 protein increased; the relative activity of caspase-3 protein in the normal serum group was 2.48±0.63, but in the three burn serum treatment groups, the relative caspase-3 activities were 3.34±0.79, 6.40±1.75 and 9.45±2.56 at 1:150, 1:100 and 1:80 concentrations, respectively. There were statistically significant differences between the three burn serum treatment groups and the normal serum treatment group, and the relative caspase-3 activity in the 1:80 burn serum treatment group was the most elevated compared with that in the normal serum group (P<0.01).

**Figure 8:**
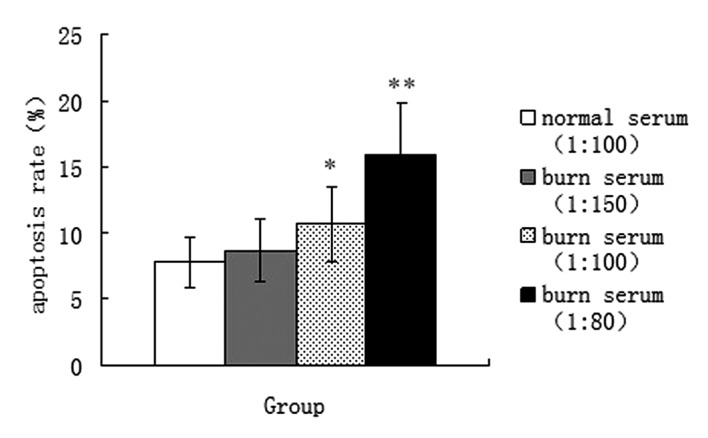
Effect of burn serum on the IEC-18 cell apoptosis rate. Data are expressed as mean ± SD. ^*^P<0.05 and ^**^P<0.01 compared with the normal serum group.

The cell apoptosis rates in the four groups of cells were compared by FCM. Fig. 8 shows that following treatment with burn serum, the cell apoptosis rate increased; the cell apoptosis rate in the normal serum group was 7.75±1.85%, but in three burn serum treatment groups, the cell apoptosis rates were 8.64±2.36, 10.7±2.86 and 15.89±3.98% at 1:150, 1:100 and 1:80 concentrations, respectively. The cell apoptosis rates in the 1:100 burn serum treatment groups were significantly different from that in the normal serum treatment group (P<0.05), and the difference was most evident between the 1:80 burn serum treatment and normal serum groups (P<0.01).

### Effect of QHBDY on caspase-3 activity and IEC-18 cell apoptosis rate

**Figure 9:**
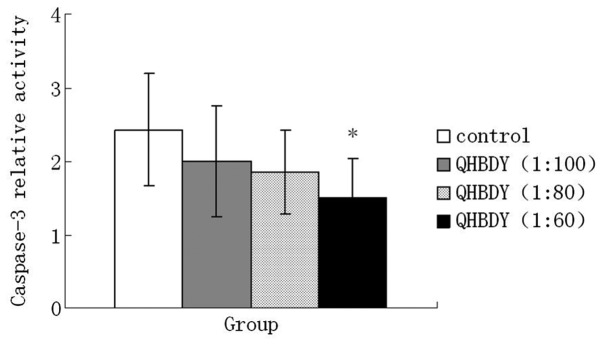
Effect of qinghuobaiduyin (QHBDY) on caspase-3 activity in IEC-18 cells. Data are expressed as mean ± SD. ^*^P<0.05 compared with the control.

Three different concentrations of QHBDY (1:100, 1:80 and 1:60) were used to treat IEC-18 cells for 24 h, and 1% saline solution was selected as control. In this experiment, the results indicate that QHBDY was able to decrease the relative activity of caspase-3. There was a statistically significant difference in caspase-3 activity between the 1:60 treatment group and the control (P<0.05; Fig. 9).

**Figure 10:**
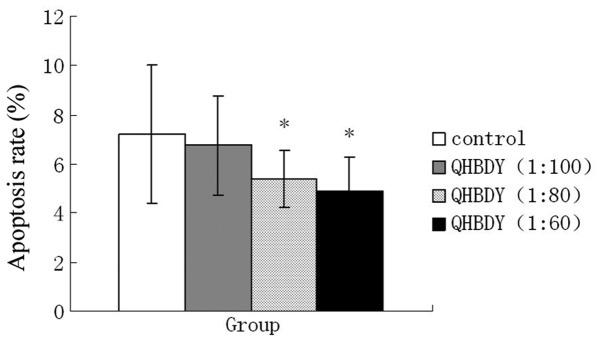
Effect of qinghuobaiduyin (QHBDY) on IEC-18 cell apoptosis rate. Data are expressed as mean ± SD. ^*^P<0.05 compared with the control.

As shown in Fig. 10, treatment with QHBDY decreased the IEC-18 cell apoptosis rates; the cell apoptosis rate in the 1:80 and 1:60 treatment groups were statistically significantly different from the control (P<0.05).

### Effect of co-treatment with QHBDY and burn serum on caspase-3 activity and apoptosis rate

**Figure 11:**
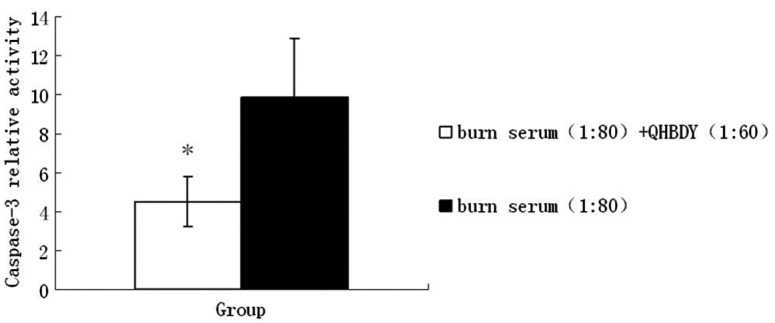
Effect of co-treatment of qinghuobaiduyin (QHBDY) and burn serum on caspase-3 activity in IEC-18 cells. Data are expressed as mean ± SD. ^*^P<0.05 compared with the burn serum group.

**Figure 12:**
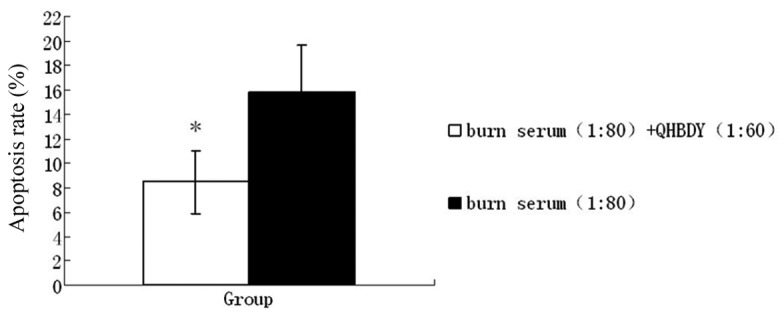
Effect of co-treatment of qinghuobaiduyin (QHBDY) and burn serum on IEC-18 cell apoptosis rate. Data are expressed as mean ± SD. ^*^P<0.05 compared with the burn serum group.

According to the experimental results of caspase-3 relative activity and IEC-18 apoptosis rate, burn serum at a concentration of 1:80 and QHBDY at a concentration of 1:60 were selected to treat IEC-18 cells together for 24 h. The results show that compared with the burn serum treatment group, the caspase-3 relative activity in the co-treatment group decreased and there was a statistically significant difference between the two groups (P<0.05; Fig. 11). The cell apoptosis rate was also statistically different between the co-treatment group and the burn serum treatment group (P<0.05); the cell apoptosis rate in the co-treatment group was lower than that in the burn serum treatment group (Fig. 12).

### Effect of co-treatment with QHBDY and burn serum on Hsp70 expression at the protein level

**Figure 13:**
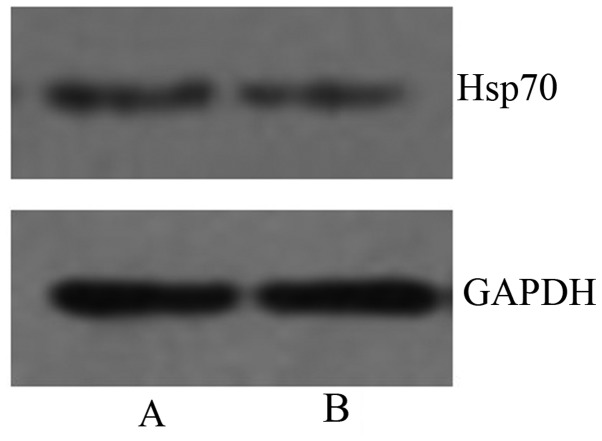
Hsp70 and GAPDH expression in IEC-18 cells. (A) Co-treatment group, (B) burn serum group. Hsp70, heat shock protein 70.

**Figure 14:**
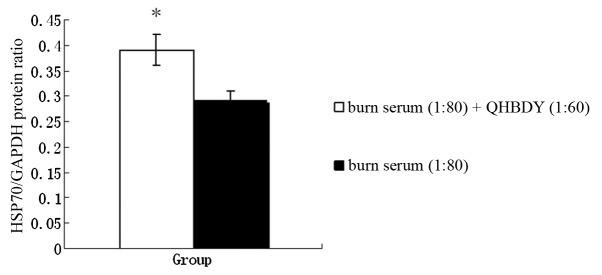
Effect of co-treatment of qinghuobaiduyin (QHBDY) and burn serum on Hsp70 protein expression in IEC-18 cells. Data are expressed as mean ± SD. ^*^P<0.05 compared with the burn serum group. Hsp70, heat shock protein 70.

After treatment with burn serum and QHBDY for 24 h, the IEC-18 cells were collected and protein was extracted in order to perform western blot analysis. GAPDH was used for quantification. The western blot images are shown in Fig. 13. The relative optical density of Hsp70 was 0.29±0.02 in the burn serum group and 0.39±0.03 in the co-treatment group. There was a statistically significant difference between the two groups (P<0.05; Fig 14).

## Discussion

The probability of infection increases greatly due to burn injury, particularly large-area burn injury. This makes the gastrointestinal tract, which is the largest bacterial storage pool in the body, particularly important. Therefore, investigation of how to reduce the degree of burn injury of the intestinal mucosa in the early stage and protect intestinal mucosal barrier function has become an important area of research. In recent years, studies of burn injury to intestinal mucosa have increasingly focused on apoptosis (1,3).

QHBDY consists of *Astragalus membranaceus*, *Lonicera japonica*, *Scutellaria baicalenis* Georgi, *Ophiopogon japonicus*, *Rheum rhabarbarum*. In previous studies, clinical and scientific research data have shown the how the opsonization function of QHBDY affects the humoral and cellular immunity of severely burned patients and its inhibitory effect on the expression of inflammatory mediators in the inflammatory response (14,15). Astragalus significantly inhibits the increase in tumor necrosis factor-α (TNF-α) levels and neuronal apoptosis in the brain tissue in rats with focal cerebral ischemia-reperfusion (16,17). Ophiopogon extract has roles in the prevention of apoptosis, proliferation and reduction of intercellular adhesion molecule-1 expression (18). In neural cell primary culture studies, it was observed that Ophiopogon injection exerted a neuroprotective effect via the prevention of apoptosis (19–22). These anti-apoptotic effects for certain tissues and organs may be attributed to certain components in QHBDY. The current study focused on the protective effect of QHBDY against intestinal mucosal apoptosis.

In order to study the anti-apoptotic effect of QHBDY, a series of experiments were carried out *in vivo* and *in vitro*. An animal model comprising severely burned rats was constructed, and these rats were divided into two categories: the burned and treatment groups. The treatment group was subdivided into three groups according to the dosage of QHBDY. The intestinal mucosal cell apoptosis rate, the expression of Hsp70 and caspase-3 was analyzed at 6, 12, 24 and 48 h after treatment. The TUNEL method was used to evaluate the intestinal mucosal apoptosis rate. It was observed that the apoptosis rates in the 1 ml/100 g and 1.5 ml/100 g groups were lower than in the burned group at 6, 12, 24 and 48 h. This suggests QHBDY was able to downregulate intestinal mucosal cell apoptosis to exert its protective effect. In addition, tissues from the small intestine were collected for immunohistochemical analysis to compare Hsp70 and caspase-3 expression at the protein level. The results showed that the expression of Hsp70 in the 1.5 ml/100 g group was higher than that in the burned group, and the expression of caspase-3 in the 1.5 ml/100 g group was lower than the burned group. These results further demonstrate the anti-apoptotic effect of QHBDY.

The anti-apoptotic role of QHBDY in cells was also investigated *in vitro*. Burn serum was added to IEC-18 cells to model the state of burning. According to our previous experiments, a drug concentration of 1:60 and a time point of 24 h were selected for the treatment of IEC-18 cells together with burn serum. The cell apoptosis rate, protein expression of Hsp70 and caspase-3 activity were analyzed prior to and following drug treatment. FCM results showed that the cell apoptosis rate in the drug treatment group was lower than in the burn serum group. This illustrated the anti-apoptotic effect of QHBDY. From western blot experiments, it was observed that the level of Hsp70 protein expression was higher in the drug treatment group than in the burn serum group. Caspase-3 activity decreased following treatment with QHBDY. These results further demonstrate the anti-apoptotic role of QHBDY in a cell model.

Therefore, it may be concluded that QHBDY may play an important role in the prevention of apoptosis at the animal and cellular levels.

## Abbreviations

QHBDY: qinghuobaiduyin
HSP: heat shock protein
FCM: flow cytometry
